# Brain imaging and machine learning reveal uncoupled functional network for contextual threat memory in long sepsis

**DOI:** 10.1038/s41598-024-79259-5

**Published:** 2024-11-12

**Authors:** Joshua J. Strohl, Joseph Carrión, Patricio T. Huerta

**Affiliations:** 1 Laboratory of Immune and Neural Networks, Feinstein Institutes for Medical Research, 350 Community Drive, Manhasset, NY 11030 USA; 2 Elmezzi Graduate School of Molecular Medicine at Northwell Health, 350 Community Drive, Manhasset, NY 11030 USA; 3 Department of Molecular Medicine, Zucker School of Medicine at Hofstra/Northwell, 500 Hofstra Blvd, Hempstead, NY 11549 USA

**Keywords:** Infection, Infectious diseases, Inflammation, Neuroimmunology, Diseases of the nervous system, Learning and memory, Neural circuits, Neuroimmunology, Immunology, Neuroscience

## Abstract

Positron emission tomography (PET) utilizes radiotracers like [^18^F]fluorodeoxyglucose (FDG) to measure brain activity in health and disease. Performing behavioral tasks between the FDG injection and the PET scan allows the FDG signal to reflect task-related brain networks. Building on this principle, we introduce an approach called behavioral task–associated PET (beta-PET) consisting of two scans: the first after a mouse is familiarized with a conditioning chamber, and the second upon recall of contextual threat. Associative threat conditioning occurs between scans. Beta-PET focuses on brain regions encoding threat memory (e.g., amygdala, prefrontal cortex) and contextual aspects (e.g., hippocampus, subiculum, entorhinal cortex). Our results show that beta-PET identifies a biologically defined network encoding contextual threat memory and its uncoupling in a mouse model of long sepsis. Moreover, machine learning algorithms (linear logistic regression) and ordinal trends analysis demonstrate that beta-PET robustly predicts the behavioral defense response and its breakdown during long sepsis.

**Supplementary Information:**

The online version contains supplementary material available at 10.1038/s41598-024-79259-5.

## Introduction

Positron emission tomography (PET) is a well-established imaging technique in which compounds labeled with positron-emitting radioisotopes are used as molecular probes to image and measure physiological processes in health and disease^1,2^. For instance, the presence and severity of cancers, cardiovascular disease, and brain disorders are routinely probed with PET scans^1–3^. The commonly used radiotracer [^18^F]fluorodeoxyglucose (FDG) allows for quantitative measurements of glucose uptake, given that FDG PET generates detailed patterns of glucose transport into cells across the whole body. Within the brain, these metabolic patterns are interpreted as nodes of neural activity that make up functional networks^4^. Besides FDG, other radiotracers can target different biological processes and have been deployed in a wide range of applications, from diagnosing and monitoring disease to exploring fundamental processes in healthy and diseased states. Gallium-68-labeled prostate-specific membrane antigen ligand 11 ([^68^Ga]-PSMA-11) has become a preferred imaging modality for prostate cancer^5^. Amyloid PET tracers such as Pittsburgh compound B ([^11^C]-PIB), [^18^F]florbetapir, [^18^F]flutemetamol, and [^18^F]florbetaben are collectively used as diagnostic tools for Alzheimer’s disease^6,7^. The radionuclides [^18^F]fluoropropyl-trimethoprim ([^18^F]FPTMP), 2-[^18^F]F-ρ-aminobenzoic acid ([^18^F]FPABA), and 2-[^18^F]-fluorodeoxysorbitol (FDS) can visualize and quantify bacterial infections in the body^8–11^.

FDG PET has been widely applied to brain diseases in both clinical^12–15^ and preclinical settings^16,17^. While much has been learned from these studies, using FDG PET in conjunction with behavioral assays remains underutilized. Notably, after an FDG injection, there is a time window when behavioral tasks can be performed so that the FDG signal may reflect the brain functional network associated with the task^17^. Therefore, by combining FDG PET with behavioral tasks, the nodes engaged by the task can be examined in the brain in health and disease.

Raw brain PET data are often analyzed with an approach called statistical parametric mapping (SPM), which compares two experimental groups by performing thousands of Student’s t tests on a voxel-by-voxel basis across the brain^18^. SPM produces statistically defined clusters of contiguous voxels, and the brain regions that share a space with the statistically significant voxels are reported. Recently, multivariate methods have been developed for finding networks of correlated activity throughout the brain. One such method, called ordinal trends (ORT) canonical variates analysis, applies supervised principal component (PC) analysis across PET datasets that involve multiple conditions within the same subjects. The PC, or combination of PCs, that leads to the greatest variance within each subject across conditions is identified and its expression is calculated for each subject^4,19,20^.

Trace threat conditioning (formerly fear conditioning^21^) is a behavioral paradigm in which a subject is presented with a conditioned stimulus (e.g., a tone) paired with an unconditioned stimulus (e.g., a mild foot-shock) separated by a temporal interval known as the trace^22,23^. Acquisition of the aversive memory causes the subject to associate the conditioned stimulus (tone) as well as the context (conditioning chamber) with the unconditioned stimulus^24^. Subsequent presentations of the tone or context elicit a defense response, the acquisition and expression of which engage the amygdala and prefrontal cortex^24–28^, which are considered the neural substrate for threat memory. In addition, temporal and contextual elements of threat memory are encoded by the hippocampus and associated regions^22,25,29,30^. These structures, which are critically important in the healthy brain, are highly susceptible to conditions that involve inflammation^16,31^.

Sepsis, defined as a life-threatening organ dysfunction caused by a dysregulated response to infection^32^, is one of the most frequent causes of death worldwide. Sepsis triggers a cascade of inflammatory responses as the body attempts to fight infection^33,34^. This can lead to symptoms like fever, elevated heart rate, and rapid breathing. If untreated or poorly treated, this overwhelming inflammatory response can impact multiple organs, including the brain, lungs, heart, kidneys, liver and the gastrointestinal tract^34,35^. Indeed, long-term survivors often experience chronic immune dysregulation, brain fog, and cognitive impairment long after the initial infection^35–42^. We have termed this critical but understudied condition ‘long sepsis’ (LS) and have developed a mouse model of LS^43,44^. While lasting memory deficits in LS are documented in both clinical and preclinical studies^44–48^, the underlying biological processes from sepsis to brain dysfunction to memory loss remain poorly understood. Septic shock triggers a massive inflammatory response that includes the release of cytokines (e.g., TNF, IL-1β, IL-6, HMBG1) into the bloodstream^34^. It has been proposed that these cytokines can cross the blood-brain barrier, activating microglia (which release further inflammatory mediators) and creating a so-called neuroinflammatory environment that can disrupt neuronal function and synaptic plasticity, crucial for memory formation^48,49^. Additionally, sepsis-related factors may compromise the integrity of the blood-brain barrier directly, allowing immune cells and other potentially harmful substances to infiltrate the brain and trigger brain dysfunction^48,49^. Although it is imperative to study the cellular and molecular factors linking LS to cognitive impairment, a systems-level approach can provide much needed insight^47^. While behavioral and anatomical results have indicated that the hippocampus is affected by LS, the brain-wide neural networks involved have not yet been fully identified^43,44^.

This study examines the functional networks of the brain in a mouse model of chronic inflammation resulting from LS. We introduce a technique called behavioral task–associated PET (beta-PET) consisting of two FDG PET scans taken across different phases of the behavioral task. The first scan is done immediately after the animal is familiarized to the behavioral apparatus (e.g., the conditioning chamber) and it represents the baseline condition. The second scan occurs after the animal undergoes the recall of contextual threat. The mouse is subjected to associative threat conditioning in the interval between the first and second scans. An important feature of beta-PET is the focused scrutiny of brain regions that encode threat memory (e.g., amygdala and prefrontal cortex) as well as its contextual elements (e.g. hippocampus and associated areas), such that the FDG PET signals from the first scan are subtracted from those of the second scan. The differential signal from beta-PET isolates the PET readout associated to the behavioral task and reduces the influence of metabolic activity that is not relevant to the task in question. Our results show that beta-PET identifies a biologically defined functional network for contextual threat memory. Moreover, using machine learning tools and ORT analysis, we determine that the brain network based on beta-PET robustly predicts the behavioral defense response and its breakdown in a mouse model of LS.

## Results

**Fig. 1:**
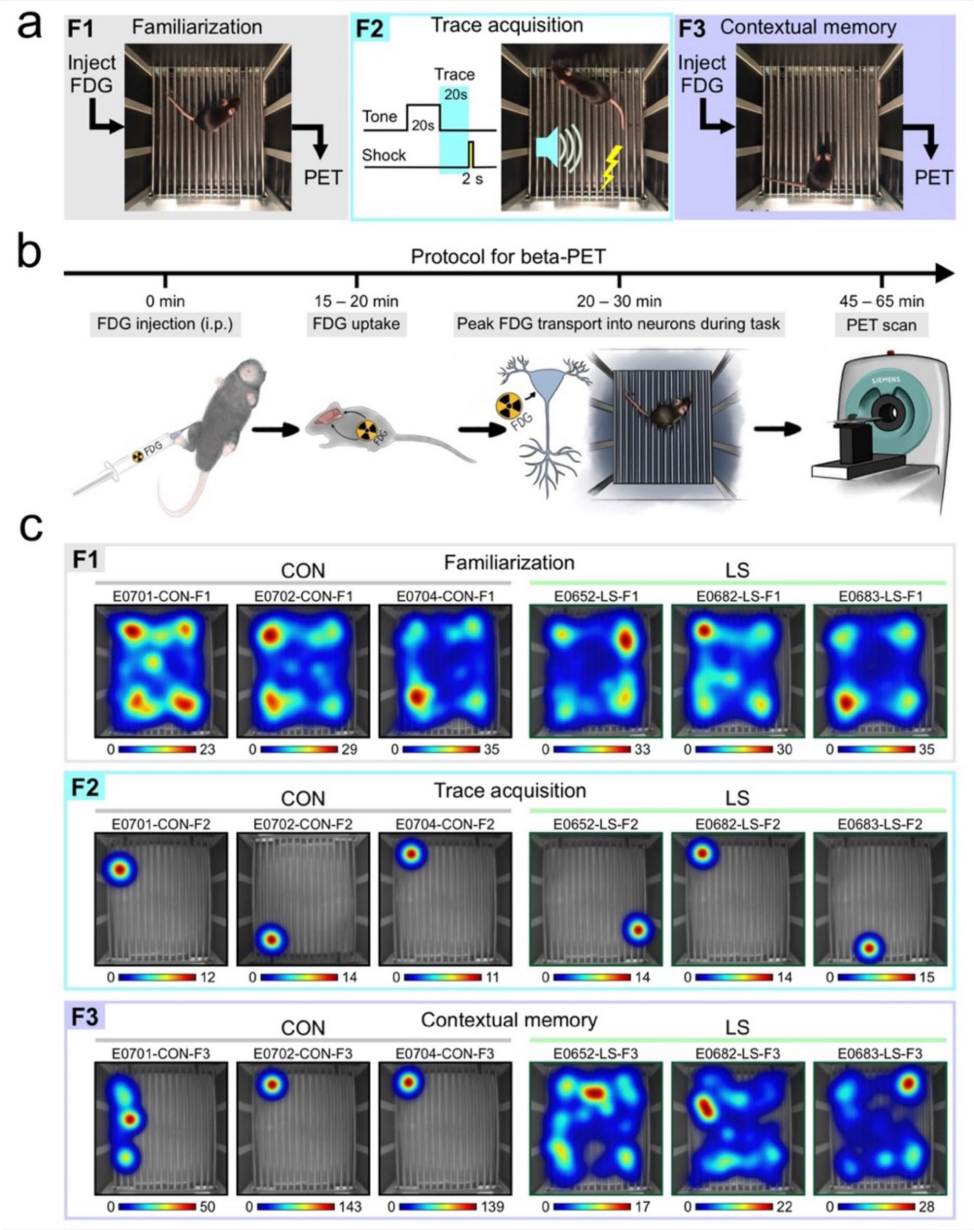
Trace threat conditioning combined with FDG PET. (**a**) Experimental design over the course of 3 days. On day 1, mice are injected with FDG, placed into the conditioning chamber for familiarization (F1), and subjected to a PET scan. On day 2, mice are exposed to trace acquisition (F2), consisting of a 20-s tone, a 20-s trace interval, and a 2-s foot-shock, repeated 3x. On day 3, mice are injected with FDG, returned to the conditioning chamber to test contextual memory (F3), and subjected to a second PET scan. (**b**) Protocol for beta-PET highlighting the time intervals of FDG injection, uptake, peak transport into neurons (which coincides with the behavioral task), and PET scan. (**c**) Representative heatmaps depicting mouse occupancy during F1 (*top*), F2 (*middle*) and F3 (*bottom*) for 3 control mice (CON, *left*) and 3 long sepsis mice (LS, *right*). The F1 heatmaps are drawn for the whole session (15 min), the F2 heatmaps for the last 30 s of trace acquisition, and the F3 heatmaps for the last 5 min of the contextual memory test. Heat scales are in seconds.

We sought to understand how chronic inflammation affects the brain using a mouse model of long sepsis. Experimental mice (termed LS henceforth) were subjected to cecal ligation and puncture (CLP), whereas control mice (termed CON henceforth) underwent sham surgery. All animals were given a period of ~ 6 weeks to recover from the surgery. Trace threat conditioning took place over the course of 3 days (Fig. 1a); the first and third days included FDG PET scans. On day 1, mice were injected with FDG and left undisturbed for 20 min to allow time for tracer uptake (Fig. 1b). Importantly, the peak FDG transport through the cell membranes of brain neurons occurs at 20–30 min post-injection^50–52^, which was precisely the time interval in which the animals were exposed to the task (Fig. 1b). Mice were placed in the conditioning chamber for a familiarization session (F1) (Fig. 1a, c), after which they were anesthetized and subjected to the first scan (45–65 min post-injection) (Fig. 1b). On day 2, mice underwent trace acquisition (F2), which consisted of 3 repetitions of a tone (80 dB, 5 kHz, 20-s long), trace interval (20 s) and a mild foot-shock (1 mA, 2-s long) (Fig. 1c). On day 3, mice were tested for contextual memory (F3) (Fig. 1a, c), with FDG injections, exposure to the conditioning chamber, and second scan occurring exactly as on day 1.

**Fig. 2:**
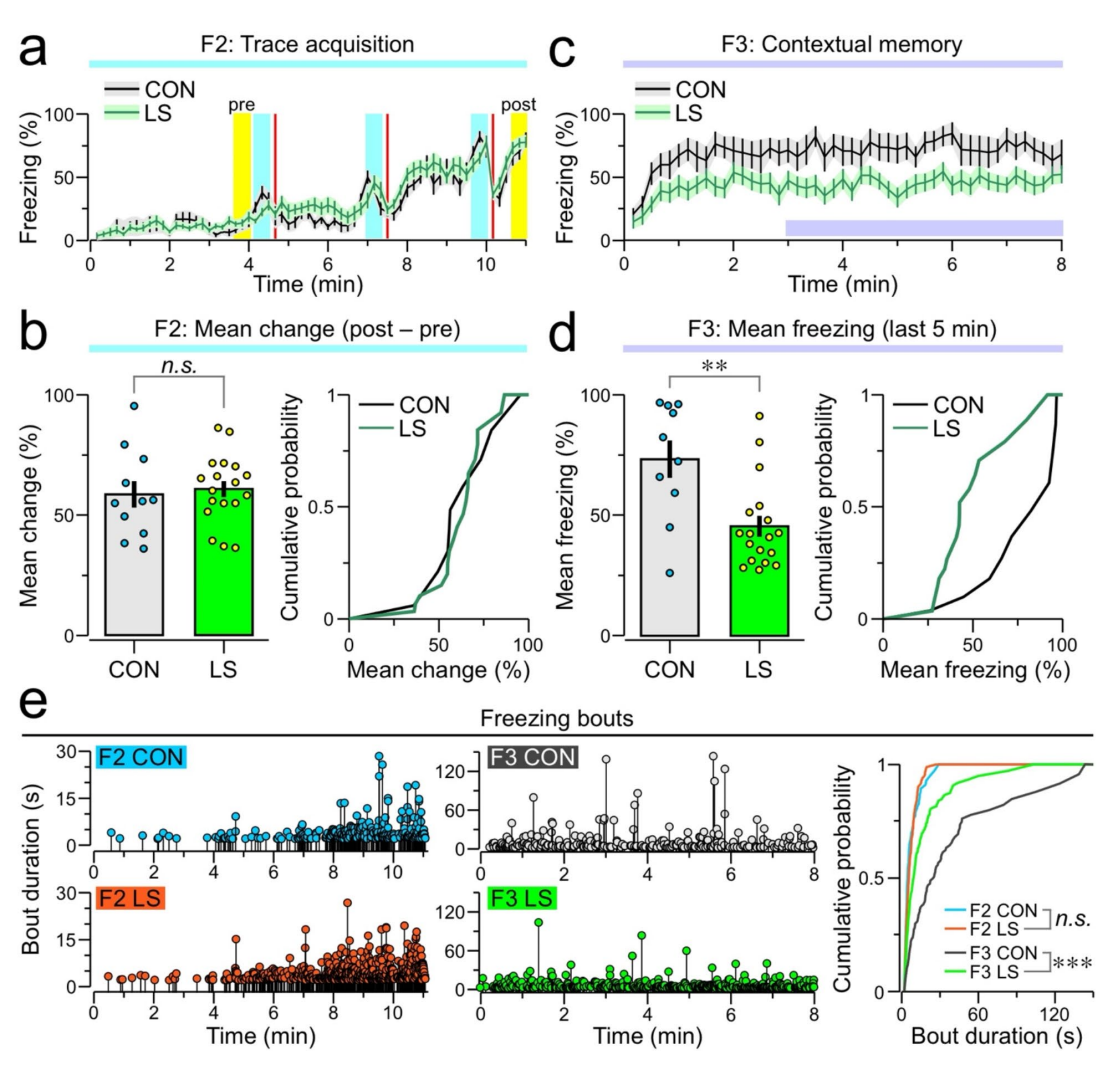
Freezing analysis of trace threat conditioning. (**a**) Time-series showing the average percent freezing (mean ± SEM) displayed by control (CON = 10) and long sepsis (LS = 18) groups on F2. The labels ‘pre’ and ‘post’ indicate the 30-s period before the initial tone–shock pairing and the last 30-s period, respectively. For each mouse, freezing was summed over a 10-s epoch and expressed as a percentage, the process was repeated for all sequential epochs, and the individual values were averaged across all animals in the group. (**b**) *Left*, bar graph showing the mean change in freezing (post – pre periods) during F2 for CON and LS mice (each dot represents a mouse); *n.s.*, non-significant, *P* = 0.72 (t test). *Right*, mean change expressed as cumulative distributions (n.s., *P* = 0.82, Kolmogorov-Smirnov [KS] test). (**c**) Time-series showing F3 freezing (mean ± SEM), using 10-s epochs. (**d**) *Left*, bar graph showing F3 freezing (mean ± SEM), during the last 5 min of the contextual memory test, for CON and LS mice (each dot represents a mouse); **, *P* = 1.98 × 10^-3^ (t test). *Right*, mean F3 freezing as cumulative distributions (**, *P* = 5.9 × 10^-3^, KS test). (**e**). *Left*, lollipop plots showing the start time and duration of each individual freezing bout during F2 and F3. *Right*, cumulative probability curve for bout duration in each group across F2 and F3; ***, *P* = 4.49 × 10^-4^ (KS test).

Assessment of the defense response was accomplished by computing freezing during the different phases of trace threat conditioning. For each mouse, freezing was summed over a 10-s epoch and expressed as a percentage, the process was repeated for all sequential epochs, and the individual values were averaged across all animals in the group^22^. F2 analysis reveals that both CON (*n* = 10) and LS (*n* = 18) groups have similar increases in freezing, measured from the 30-s period before the initial tone–shock pairing (labeled ‘pre’ in Fig. 2a) to the last 30-s period on F2 (labeled ‘post’ in Fig. 2a). Analysis of the mean change in freezing (e.g., post – pre periods) reveals similar values in both groups (mean change ± SEM, CON = 58.56 ± 5.49%, LS = 60.74 ± 3.35%, *T* = 0.36, *P* = 0.72, t test). During F3, LS mice freeze significantly less when compared to CON mice (Fig. 2c,d) (mean freezing for the last 5 min on F3, CON = 73.11 ± 7.64%, LS = 45.44 ± 4.27%, *T* = 3.44, *P* = 1.98 × 10^− 3^, t test). We also find that the CON group exhibits significantly longer freezing bouts during F3 (Fig. 2e, *D* = 0.145, *P* = 4.49 × 10^− 4^, Kolmogorov-Smirnov test). Of the 28 mice scanned, there were 4 animals for which the scan failed due to technical issues, yielding 9 CON and 15 LS scans.

**Fig. 3:**
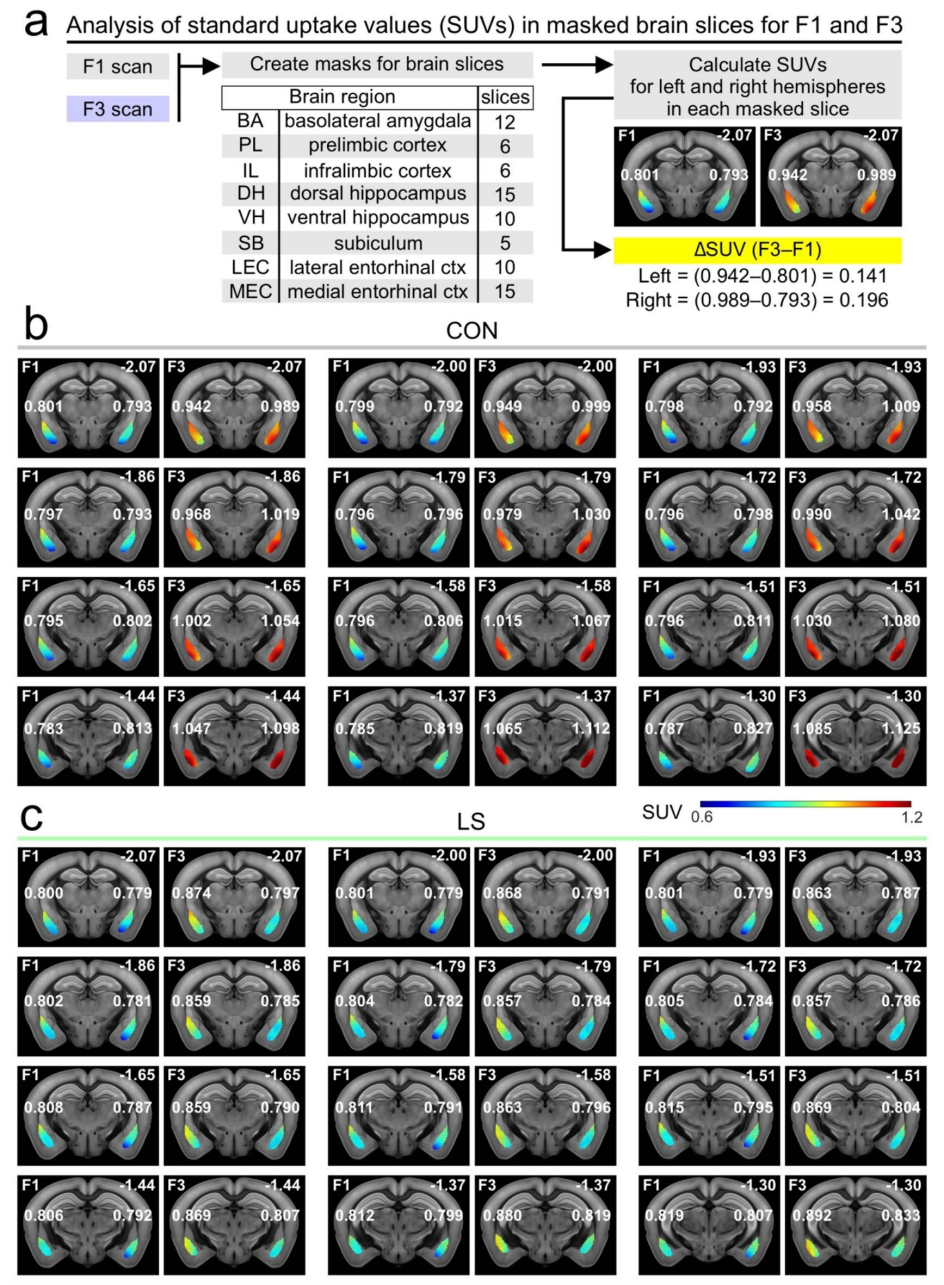
Demonstration of the beta-PET approach. (**a**) Workflow for beta-PET. Masks are drawn into coronal brain slices (see Methods) and the number of slices (for the 8 brain regions) is indicated. Standard uptake values (SUVs) are calculated for the masks, and the values for F1 are subtracted from the values for F3 to obtain ΔSUV(F3–F1). (**b**) Representative scans from a CON mouse showing the 12 slices used for BA. The F1 and F3 scans are shown side by side for each slice, with the heatmap scaled to the SUV. The value of each hemisphere (within each slice) is indicated. The number at the top right of each slice represents the distance from bregma (in mm). (**c**) Representative scans for the BA analysis in a LS mouse, similar as in (**b**).

**Fig. 4:**
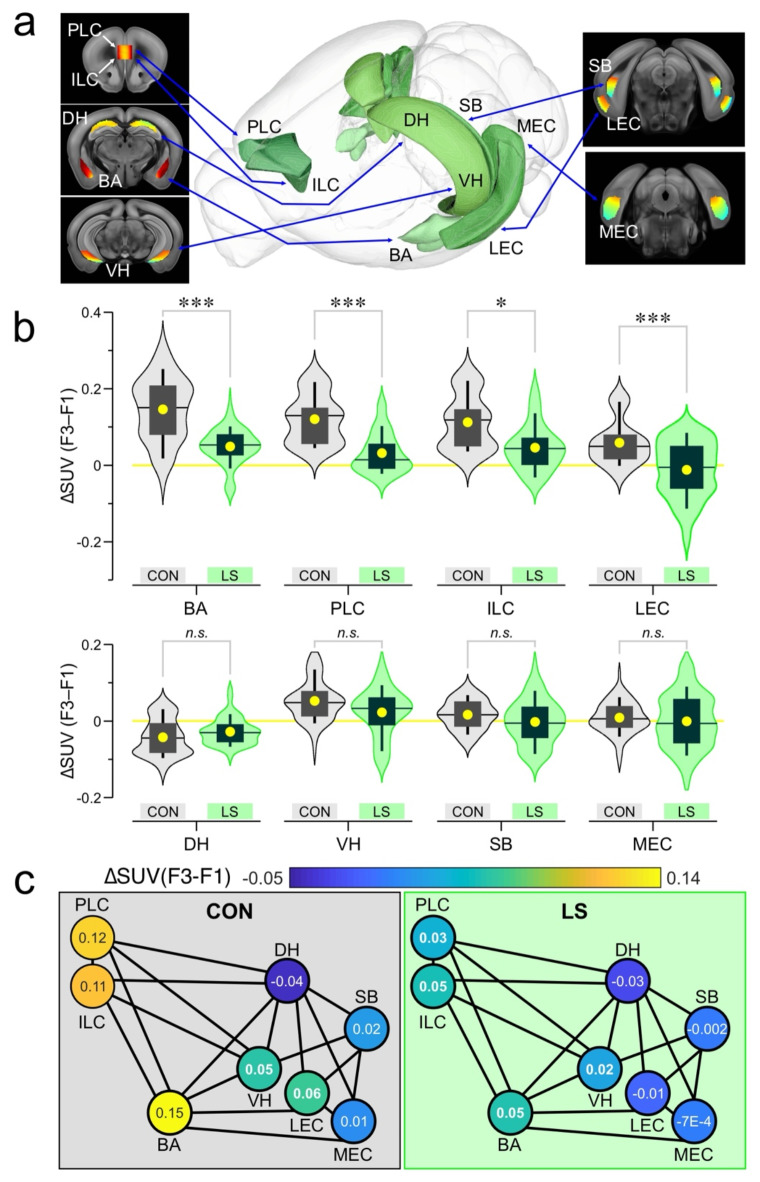
Beta-PET applied to the functional network encoding threat memory. (**a**) Diagram showing 3D renderings of the brain regions under study, alongside coronal sections showing SUVs averaged across all CON mice during F3. Abbreviations: BA, basolateral amygdala; DH, dorsal hippocampus; ILC, infralimbic cortex; LEC, lateral entorhinal cortex; MEC, medial entorhinal cortex; PLC, prelimbic cortex; SB, subiculum; VH, ventral hippocampus. (**b**) Violin plots show kernel smooth distributions of ΔSUV data. Box-and-whisker plots show mean (circle), median (line), inter-quartile range (box), and 10-90 distribution (whiskers). *Top*, brain regions with significant differences: BA, ***, *P* = 2.57 × 10^-5^; PLC, ***, *P* = 1.3 × 10^-4^; ILC, *, *P* = 0.014; LEC, ***, *P* = 2.8 × 10^-4^; statistics with linear mixed model. *Bottom*, brain regions with non-significant (*n.s.*) differences: DH, *P* = 0.27; VH, *P* = 0.19; SB, *P* = 0.34, MEC, *P* = 0.68. (**c**) Network map showing the ΔSUV for each brain region averaged across all mice from CON (*left*) and LS (*right*) groups; each node represents a brain region. The heatmap for each node is scaled to the ΔSUVs, with the average ΔSUV indicated by the number shown within each node.

To elucidate how the brain networks that encode aversive memory (including its contextual aspects) fail during LS, we isolated the FDG PET signals associated with the behavioral task. There is strong agreement on the neural substrates for threat memory^24–30^, which allowed us to make informed decisions about which brain regions to select for beta-PET. Using coronal sections, we analyzed each slice by drawing a mask around a region-of-interest, independently for the left and right hemispheres, except for the prefrontal cortex regions which were analyzed as a single object for each slice^53,54^ (Fig. 3a). We then calculated the standard uptake value (SUV) for each masked slice and subtracted the F1 SUVs from the F3 SUVs to obtain ΔSUVs (Fig. 3a). Given the nested nature of analyzing multiple slices and hemispheres for each mouse, we used general linear–mixed–model (LMM) statistics to account for the multiple values arising from each mouse. Using this differential approach in CON (Fig. 3b) and LS (Fig. 3c) groups, we examined the basolateral amygdala (BA), prelimbic cortex (PLC), infralimbic cortex (ILC), dorsal hippocampus (DH), ventral hippocampus (VH), subiculum (SB), lateral entorhinal cortex (LEC), and medial entorhinal cortex (MEC)^55,56^ (Fig. 4a, Supplementary Data 1). We chose BA, PLC, and ILC because these regions are the core neural nodes that encode threat conditioning^25–29,56–61^. As our study focused on the contextual memory association in threat conditioning, we chose regions that encode spatial context, such as DH, VH, MEC, LEC, and SB^25,29–31,62–71^. Figure 4b shows significantly abrogated responses in LS mice (*n* = 15), when compared to CON mice (*n* = 9) for BA, PLC, ILC and LEC (BA: CON = 0.15 ± 5.7 × 10^− 3^ ΔSUV, LS = 0.049 ± 2.5 × 10^− 3^, *F* = 18, *P* = 2.57 × 10^− 5^; PLC: CON = 0.12 ± 8.5 × 10^− 3^, LS = 0.032 ± 5.5 × 10^− 3^, *F* = 15.55, *P* = 1.3 × 10^− 4^; ILC: CON = 0.11 ± 9.5 × 10^− 3^, LS = 0.046 ± 6.2 × 10^− 3^, *F* = 6.21, *P* = 0.014; LEC: CON = 0.059 ± 4.3 × 10^− 3^, LS = -0.012 ± 4.3 × 10^− 3^, *F* = 13.4, *P* = 2.8 × 10^− 4^; statistics with LMM). Figure 4b also shows no significant differences between groups for DH, VH, SB and MEC (DH: CON = -0.043 ± 2.9 × 10^− 3^ ΔSUV, LS = -0.028 ± 1.7 × 10^− 3^, *F* = 1.21, *P* = 0.27; VH: CON = 0.052 ± 3.9 × 10^− 3^, LS = 0.023 ± 3.6 × 10^− 3^, *F* = 1.72, *P* = 0.19; SB: CON = 0.016 ± 4.0 × 10^− 3^, LS = -2.4 × 10^− 3^ ± 4.8 × 10^− 3^, *F* = 0.93, *P* = 0.34; MEC: CON = 8.6 × 10^− 3^ ± 2.6 × 10^− 3^, LS = -7.5 × 10^− 4^ ± 3.3 × 10^− 3^, *F* = 0.18, *P* = 0.68; LMM). When the mean ΔSUVs for each group are shown in a network map (Fig. 3c), the functional responses can be clearly visualized in CON mice (enhanced glucose uptake in BA, PLC, and ILC) as well as their striking absence in the LS group.

**Fig. 5:**
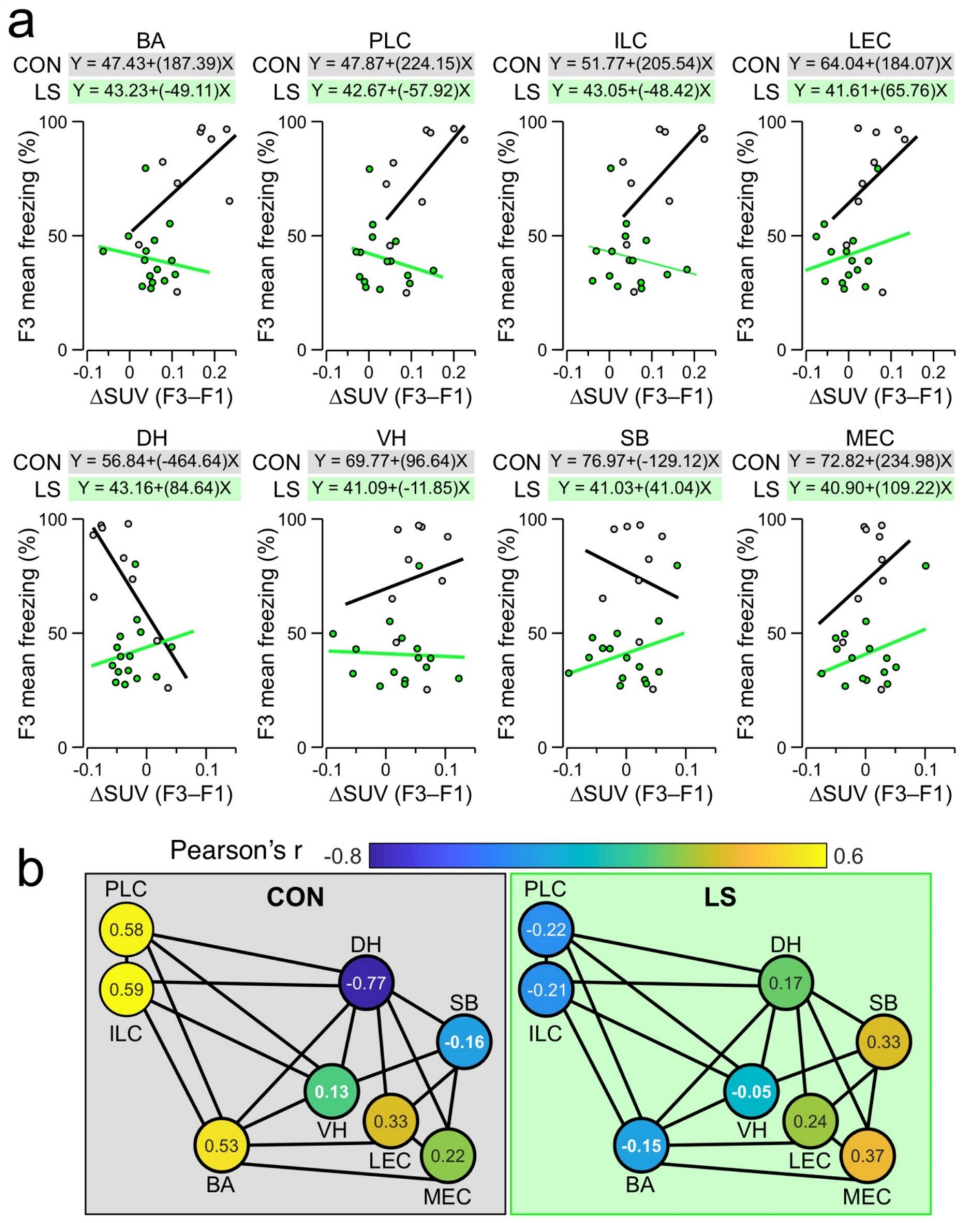
Correlations between beta-PET and contextual threat memory. (**a**) Scatter plots showing correlations of ΔSUV for each brain region against the mean freezing during the last 5 min of F3. Each dot represents an individual mouse. For each plot, the grey line shows the linear fit for CON mice, and the green line is the linear fit for LS mice. The equations describing the linear fits are shown above the plots. Abbreviations same as in Fig. 3. (**b**) Network map showing the Pearson’s *r* values describing the correlation of ΔSUVs for each brain region with behavior, averaged across all mice from each group (each node represents a brain region). *Left*, average Pearson’s *r* values for CON mice. *Right*, average Pearson’s r values for LS mice. The heatmap for each node is scaled to the Pearson’s *r* values, with the average Pearson’s *r* indicated by the number shown within each node.

We next examined the correlations between ΔSUV(F3–F1) and contextual threat memory. For each mouse, we took the ΔSUV value from each brain region under analysis as the independent variable and plotted it against the behavioral readout (average freezing during the last 5 min on F3) as the dependent variable. We fitted the CON and LS datasets with linear regressions and calculated the Pearson’s correlation coefficient (*r*) for each curve (Fig. 5a). CON mice have the strongest positive correlations between ΔSUV and freezing in ILC (*r* = 0.59), PLC (*r* = 0.58), and BA (*r* = 0.53) as well as a strong negative correlation in DH (*r* = − 0.77). Notably, LS mice exhibit a contrasting pattern of correlations when compared to CON mice, with negative correlations in ILC (*r* = -0.21), PLC (*r* = − 0.22), and BA (*r* = − 0.15) and a positive correlation in DH (*r* = 0.17). Surprisingly, of all the brain areas examined, LS mice have the strongest positive correlation in MEC (*r* = 0.37), although this correlation is of lower magnitude than those found in the key areas of CON mice. When the Pearson’s *r* values for each group are shown in a network map (Fig. 5b), the correlated responses can be clearly visualized in CON mice (elevated correlations in BA, PLC, and ILC) as well as the negative values in the LS group.

**Fig. 6:**
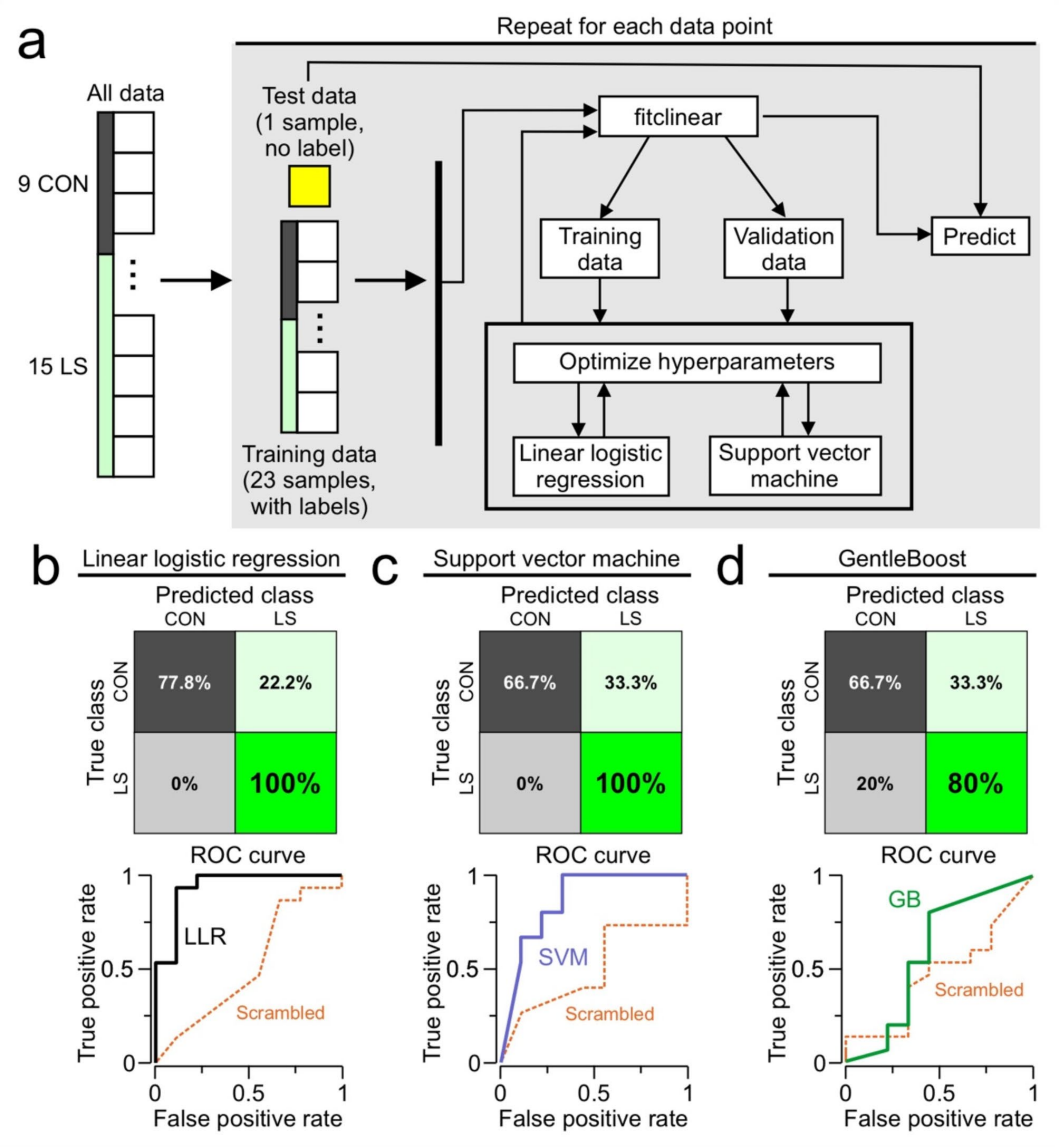
Machine learning predicts class based on beta-PET. (**a**) Diagram illustrating the implementation of leave-one-out cross validation. The dataset consists of 8 features and a class label for each mouse. The features are the beta-PET ΔSUVs corresponding to each brain region (regions depicted in Fig. 3), and the class label is either CON or LS. For each iteration of the cross-validation loop, one datapoint (with no class label) is chosen as the test data. The test data is excluded from training the model. The remaining labeled datapoints are used as training and validation data, and the *fitclinear* function is used to train the model and tune the hyperparameters. The trained model is used to predict the class of the test data. This loop is repeated with a different mouse assigned to the test data for each iteration, until all of the mice have been classified by the algorithm. The same leave-one-out cross validation approach is also implemented using a scalable vector machine (using the *fitcsvm* function), and GentleBoost (using the *fitcensemble* function). (**b**) *Top*, confusion matrix for the linear logistic regression. The predicted class is shown on the top and the true class is shown on the left. Treating CON as the negative class, and LS as the positive class, the true negative rate is shown in the upper left box (77.8%), the false positive rate is shown in the upper right box (22.2%), the false negative rate is shown in the lower left box (0%), and the true positive rate is shown in the lower right box (100%). *Bottom*, receiver operating characteristic (ROC) curve for the linear logistic regression. The performance of the algorithm is shown in the solid line, while the dashed line shows the performance of the algorithm when the classes and features are scrambled. (**c**) Confusion matrix and ROC curve for support vector machine, similar as in (**b**). (**d**) Confusion matrix and ROC curve for GentleBoost, similar as in (**b**).

To evaluate the efficacy of our results in identifying the chronic inflammation condition, we decided to apply a machine learning approach. Our goal was to predict the class of each mouse as either CON or LS based solely on PET data. We tested three different machine learning classification algorithms, using the ΔSUVs (of each region-of-interest) as our input features. The algorithms included linear–logistic–regression (LLR), support–vector–machine (SVM), and an ensemble-tree based method using GentleBoost. We performed these operations in MATLAB (Statistics and Machine Learning Toolbox, version R2024a, MathWorks, Inc., Natick, MA, https://www.mathworks.com/products/statistics.html) using the functions *fitclinear* (for LLR), *fitscsvm* (for SVM), and *fitcensemble* (for GentleBoost). We implemented a leave-one-out cross validation approach to account for the relatively small sample sizes of our datasets. This approach was done using a loop where one mouse was taken out for each iteration to be used as the test data. The classification model was trained on the remaining mice, with the MATLAB functions automatically splitting the data into training and validation datasets. The *fitclinear* function selected between LLR and SVM classifiers as a hyperparameter and chose an LLR, based on the training and validation data (Fig. 6a). Nevertheless, we show the results from *fitcsvm* and *fitcensemble* for comparison, although these models did not perform as well as the LLR. Treating CON as the negative class, and LS as the positive class, we found that the LLR model performed exceptionally well, giving a true negative rate of 77.8% a true positive rate of 100%. We created ROC curves for each classifier and scrambled the labels for training the models (to determine if our models fared better with the real data than the scrambled data). For the LLR, the curve with the real data showed much better classification than that for the scrambled data, which resembled that of a random classifier (Fig. 6b). We found the SVM model did not perform as well as the LLR, with a true negative rate of 66.7% and a true positive rate of 100%. The ROC curve for the real data showed better classification than for the scrambled data, but this was not as impressive as the LLR (Fig. 6c). The Gentleboost model performed the worst with a true negative rate of 66.7% and a true positive rate of 80%. The ROC curve for this dataset indicated a performance only slightly better than that of the scrambled data, indicating that this model would be an inappropriate choice for our data (Fig. 6d).

**Fig. 7:**
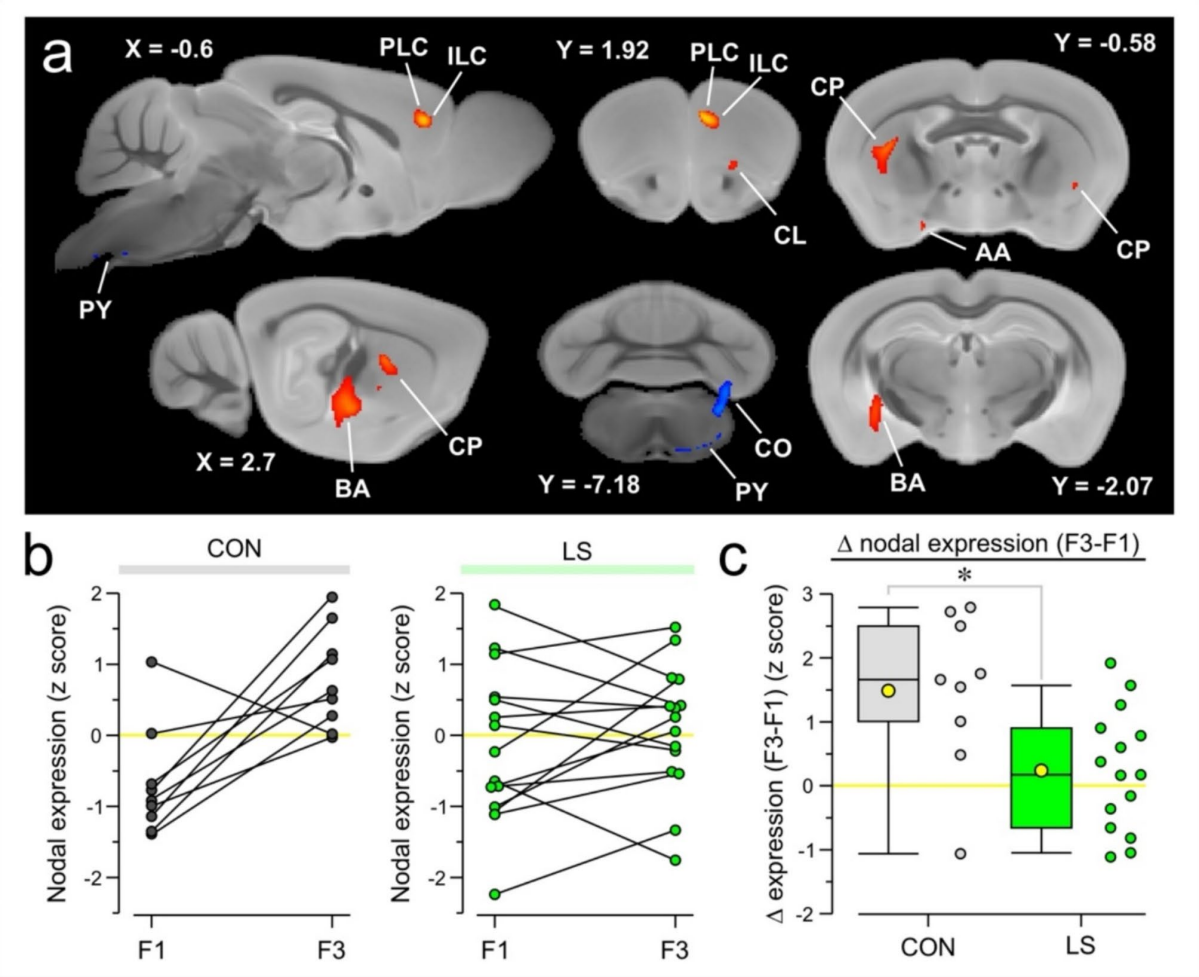
Ordinal trends analysis validates behavior-associated nodes. (**a**) Statistically significant voxels displayed over mouse brain MRI template. Ordinal trends (ORT) analysis is performed on CON mice using scans from F1 and F3, and the PC with the greatest variance across scans is identified. The principal component (PC) is bootstrapped 500 times, and the statistically significant voxels are plotted over cross-sections of a mouse brain MRI template. Voxels which increase from F1 to F3 are shown in red, while voxels that decrease from F1 to F3 are shown in blue. The distance from midline (in mm) is shown next to each sagittal slice (e.g., X = -0.6), and the distance from bregma (in mm) is shown for each coronal slice (e.g., Y = 1.92). Regions identified from the significant voxels include the prelimbic cortex (PLC), infralimbic cortex (ILC), basolateral amygdala (BA), claustrum (CL), caudate putamen (CP), anterior amygdala area (AA), copula pyramidis (CO), and the pyramidal tract (PY). (**b**) Line series plots showing the z-scored nodal expression for F1 and F3 of PC indicated in (**a**). Each pair of connected dots represents a mouse. *Left*, CON mice, *right*, LS mice. (**c**) Box-and-whisker plot of the change in nodal expression of the PC indicated in (**a**) from F1 to F3 (each dot represents a mouse). Box-and-whisker plot shows mean (square), median (line), inter-quartile range (box), and 10-90 distribution (whiskers); *, *P* = 0.02, t test.

Given the strength of our results using the beta-PET approach, we sought to validate it against an accepted multivariate approach for analyzing PET data. Our dataset consisted of two sequential conditions for each mouse, making ORT analysis an appropriate choice, which we applied on the scans of CON mice, using F1 as the first condition and F3 as the second condition. This analysis generated PCs, which were assessed on their own, and in linear combinations with each other using the Akaike information criteria. Through this approach, it was determined that one PC (PC3) had the best fit. This PC was found to exhibit a statistically significant ordinal trend using the permutation test (*P* = 0.018). The voxel weights for the ORT were evaluated using bootstrap resampling to ensure reliability, and the statistically significant voxel weights (*P* < 0.05, one tailed, 500 iterations) were plotted over a magnetic resonance imaging (MRI) template that was aligned with the PET scans. This approach found significant voxel weights in PLC, ILC, and BA, thus confirming our beta-PET approach. Interestingly, ORT analysis also identified other regions including the caudate putamen (CP), claustrum (CL), anterior amygdala area (AA), copula pyramidis (CO) which is part of the cerebellum, and pyramidal tract (PY) within the brainstem (Fig. 7a). We plotted the nodal expression of the identified PC for CON and LS mice from F1 to F3 and found that 88.9% of CON mice had an increase in nodal expression, compared to only 60% of LS mice (Fig. 7b). We then subtracted the change in nodal expression from F1 to F3, which revealed a significantly lower expression in LS mice when compared to CON mice (Fig. 7c, CON = 1.49 ± 0.41 z-score, LS = 0.24 ± 0.94, *T* = 2.62, *P* = 0.02, t test).

## Discussion

We have studied the brain metabolic patterns that are engaged in response to the contextual memory of an aversive event. Through the use of several analytical methods, we have shown that these metabolic patterns fail to activate following chronic inflammation. We have introduced the beta-PET technique (Figs. 1, 2, 3 and 4), which involves performing FDG PET scans in two phases of a behavioral task, and then using a differential region-of-interest analysis to measure the response in the chosen brain areas. Notably, the beta-PET results correlate with the behavioral readout (mean freezing during F3) (Fig. 5) and are highly accurate in differentiating between CON and LS mice with machine learning classification algorithms (Fig. 6). Furthermore, this approach has enabled us to generate a map across the multiple brain areas we examined, providing a clear visualization of the way in which LS alters the brain network responsible for encoding contextual threat memory (Fig. 4c). We have confirmed our results using ORT analysis (Fig. 7), a multivariate approach which identified brain areas overlapping with those examined with beta-PET. While the brain regions identified by ORT analysis agreed with those used in beta-PET, ORT analysis also highlighted a few regions that are unrelated to contextual threat memory. These areas include parts of the cerebellum, striatum, and brainstem. We postulate that the metabolic changes detected in these regions are a consequence of the mouse’s reduced motility during F3 rather than a cognitive process associated with contextual memory. Notably, the beta-PET approach allows us to focus on the brain regions related to the task under investigation. Thus, we think this overall approach is well suited to study a wide variety of behavioral tasks and brain disorders.

In our study, the brain areas chosen for beta-PET are essential components of the brain’s threat network, particularly the prefrontal cortex and amygdala^25,29–31,62–71^. Indeed, the change in metabolic activity in these regions is strongly correlated with the level of freezing during the contextual memory test in CON mice. Notably, not only is the change in metabolic activity smaller in LS mice, but there is also uncoupling in the correlation between metabolism and behavior. Previous studies using the LS model focused on the hippocampus as the neural substrate for cognitive impairment^43,44^, but our results demonstrate that there are broader neural abnormalities which extend to the prefrontal cortex and amygdala.

It is useful to consider the translational relevance of our preclinical findings to human sepsis survivors. While this study focuses on establishing beta-PET in a mouse model of LS, we believe it lays the groundwork for clinical research into long-term cognitive deficits, including memory impairment, following sepsis. We do not envision beta-PET as a standalone diagnostic tool for memory deficits in humans, but rather as a valuable component within a comprehensive assessment of LS. The beta-PET analysis highlights a distributed network of brain regions involved in threat memory processing, which engages a complex network rather than isolated brain regions^78^. A key challenge is differentiating sepsis-induced brain dysfunction from other neurological conditions^12–15^. We hypothesize that the distributed patterns of network activity, rather than changes in isolated brain regions, may provide a sensitive indicator. Our machine learning analysis in mice successfully distinguished between control and LS groups based solely on the distributed PET signal. This suggests that analyzing network-level alterations, as revealed by beta-PET, could provide a sensitive and specific indicator of sepsis-induced brain dysfunction in humans. For example, while Alzheimer’s disease typically exhibits focal atrophy and hypometabolism in medial temporal lobe structures^12^, sepsis might manifest as a more diffuse disruption across frontotemporal and limbic networks. Directly comparing network activity patterns in sepsis survivors with those in patients with other neurological conditions^12–15^ (e.g., Alzheimer’s, traumatic brain injury) will be essential to validate the specificity of beta-PET for detecting sepsis-related cognitive impairment.

To translate these preclinical findings to humans, we envision a modified beta-PET protocol adapted for clinical use. Similar to our mouse paradigm, human participants would undergo two PET scans: a baseline scan after FDG injection and a rest period, followed by a second scan after FDG injection and a threat memory recall session the following day, with intervening threat conditioning. The conditioning paradigm could involve pairing a visual cue with a mild wrist shock, as demonstrated in previous human studies^77^. Analyzing ΔSUV between the two scans across the whole brain would reveal task-related changes in network activity. While human studies on long-term cognitive outcomes after sepsis are sparse^30,36,72–76^, the pervasive nature of cognitive impairment in sepsis survivors underscores the urgent need for such investigations. Our preclinical findings provide a rationale and a potential methodological framework for translational research aimed at developing targeted interventions for sepsis-associated cognitive impairment.

Radiotracer imaging has broad potential to examine the multiple factors which contribute to LS. While this study focused on FDG to examine neural network activity, other radiotracers could provide additional insights on the effects of LS in the brain. It might be possible to examine whether there are lingering bacterial infections anywhere in the body, but particularly in the brain, using tracers such as [^18^F]FPTMP, [^18^F]-FPABA and FDS^8–11^.Furthermore, one can image glial activation in the brain with the tracer [^11^C]-1-(2-chlorophenyl)-N-[1-(1-methylpropyl)-2-isoquinolinyl]-3-(5-methoxy-1 H-indol-3-yl)-2-propanamine ([^11^C]PBR28)^79,80^, and the possible abnormalities in the integrity of the blood-brain barrier using [^11^C-methyl]-alpha-aminoisobutyric acid ([^11^C]AIB)^81,82^.

A limitation of this study is the use of the murine CLP model, which has recently come under heavy scrutiny as being a poor model of human sepsis^83,84^. While the CLP model is appropriate for this study, considering alternative approaches is important. The endotoxemia rodent model involves administering bacterial lipopolysaccharide (a component of the outer membrane of Gram-negative bacteria) to the animal^85,86^. This induces a systemic inflammatory response mimicking key early events of sepsis. Advantages of the endotoxemia model include its relative ease of establishment, cost-effectiveness, and reproducibility^85^. However, limitations include its short duration, focus on the initial hyperinflammatory phase, and lack of the later immunosuppressive phase often observed in patients. Unlike the CLP model, the endotoxemia model does not replicate the long-term, complex, systems-level dysregulation characteristic of polymicrobial sepsis^85^. Newly developed pneumonia-based mouse models, reflecting pneumonia’s role as a common cause of sepsis, are likely to provide crucial translational insights^87–89^. Indeed, pneumonia-induced sepsis has been shown to produce pathological conditions in the brain^87–89^. While we acknowledge the criticisms of the CLP model, we would like to point out three considerations. First, the vast majority of studies that have used the CLP model have examined the first 24–72 h after the procedure, while our model investigates survivors several weeks after recovery; indeed, the long-term sequalae of sepsis remain largely understudied in preclinical models as well as in human patients. Second, most studies using CLP have focused heavily on specific immunological dysregulations triggered by the septic shock, often offering the modification of a single immune factor as a potential therapeutic strategy. Unsurprisingly, this reductionist approach has led to several failed clinical trials in humans. In contrast, the LS model we present applies a broader systems neuroscience perspective, which is removed from the initial immunological trigger(s). Third, the Sepsis-3 definition states that sepsis is a life-threatening organ dysfunction caused by an abnormal host response to infection^39^. We think the brain dysfunction we have identified in our LS model fits smoothly into the Sepsis-3 definition and offers important translational insights. Our murine findings point to the brain’s network encoding contextual threat memory as an indicator of organ dysfunction in human sepsis survivors.

## Methods

### Ethics statement

Animal experiments were performed in accordance with the National Institutes of Health (NIH) Guidelines under protocols approved by the Feinstein Institutes for Medical Research Institutional Animal Care and Use Committee (IACUC). Our Animal Research Program is registered with the Department of Health and Human Services (DHHS), Office of Laboratory Animal Welfare (OLAW), U.S. Department of Agriculture (USDA #21R0107), Public Health Service (PHS #A3168–01) and New York State Department of Health (NYSDOH #A- 060). Efforts were performed to minimize the number of animals used and their suffering. All procedures involving experimental animals were performed in accordance with ARRIVE guidelines, reviewed and approved by the Feinstein’s IACUC, protocol code #2023-003.

### Mice

C57BL/6 mice were purchased from the Jackson Laboratory (Bar Harbor, ME) and maintained on a 12-h reverse circadian cycle (dark 9:00–21:00, light 21:00–9:00) with ad libitum access to water and chow. After arrival, mice were left undisturbed for one week prior to the start of any handling or experiments to allow for acclimation. Mice were initially housed 5 per cage. All experiments were performed during the dark phase of the circadian cycle.

### Cecal ligation and puncture procedure

At 8 weeks of age, mice underwent either CLP or sham surgery. Mice were anaesthetized using isoflurane (1.5–2.5%) and the surgical site was shaved and wiped clean with betadine solution and isopropyl alcohol. The peritoneal cavity was opened and the cecum ligated below the ileocecal valve using 4 − 0 silk suture. The cecum was then punctured using a 22G needle and ~ 1 mm of stool was extruded. The cecum was placed back into the mouse, and the incision site was closed with 6−0 Ethilon silk (Ethicon, NJ) and wound clips (Stoelting, IL). For the sham operation, mice were opened, the cecum was exposed, and the incision site was closed with no ligation or puncture made to the cecum. All mice received 0.5 mL saline resuscitation, a single dose of buprenorphine (Buprenex, 0.03 mg per kg), and a single dose of antibiotics (primaxin, Merck, Kenilworth, NJ, 0.5 mg per mouse, in 0.2 mL sterile saline) immediately following the surgery via intraperitoneal injection.

### Mouse handling

Mice were handled for 15 min per day for 3 days prior to the start of behavioral tasks, in a setting with high light. Importantly, handling occurred in a different room from where behavioral experiments were carried out.

### Trace threat conditioning

We have previously published a variant of these procedures^22,31^. Videos from all behavioral experiments were recorded using an overhead camera. The videos were saved, tracked, and analyzed using EthoVision XT (version 14, Noldus, Leesburg, VA, USA, https://www.noldus.com/ethovision-xt). The same software controlled the administration of the tones and foot-shocks. Trace threat conditioning was performed over the course of 3 days. On day 1, mice were placed in the conditioning chamber (18 cm × 18 cm × 30 cm) for a familiarization session of 10 min and returned to their home cages. On day 2, mice were placed back into the chamber and, over the course of 11 min, were subjected to three 20-s long tones (80 dB, 5 kHz), each of which was followed by a 20-s trace period and a foot-shock (1 mA, 2-s long). Contextual threat memory was tested on day 3 by returning mice to the conditioning chamber for 8 min with no tones or foot-shocks.

### Freezing analysis

The activity analysis feature in EthoVision tracked the number of pixels that changed from frame to frame and determined the amount of movement or freezing by calculating how many frames showed pixel changes. These activity scores for every video frame were imported into a custom script in MATLAB (version R2024a, MathWorks, Inc., Natick, MA, USA, https://www.mathworks.com/products/new_products/release2024a.html) which allowed the user to set a threshold for what was considered activity compared to inactivity. The percent freezing was then calculated over a 10-s interval basis, which reflected the percent of frames with inactivity over this interval. For each mouse, freezing was summed over a 10-s epoch and expressed as a percentage, the process was repeated for all sequential epochs, and the individual values were averaged across all animals in the group. The bout analysis was performed with a custom MATLAB script that used the unbinned activity data and calculated the start time and duration of each freezing bout (periods of 2 s or longer were considered as bouts).

### FDG PET

Mice were injected intraperitoneally with FDG (0.5–1.0 mCi), which was followed by a waiting period of 20 min prior to placement into the conditioning chamber during the familiarization and contextual memory sessions (Fig. 1b). The time between FDG injection and placement into the chamber (20–30 min post-injection) was chosen to allow for maximum FDG transport into brain neurons during the behavioral task. After F1 or F3, mice were anesthetized with 2.5% isoflurane and placed onto the Siemens Inveon PET scanner (Siemens SG, Munich, Germany) where anesthesia was maintained using 2% isoflurane. A 10-min emission scan followed by an 8-min transmission scan were obtained and reconstructed. The final matrix had a size of 128 × 128 × 159 mm with a pixel size of 0.78 × 0.78 × 0.8 mm.

### PET scan preprocessing

All acquired images were preprocessed using PMOD (version 3.3, PMOD Technologies Ltd., Zurich, Switzerland, https://www.pmod.com/web/) prior to further analysis. First, the brain was isolated from the rest of the body by cropping acquired images to a box of size 4.9 × 20.7 × 11.9 mm and aligned to an anatomical template which was generated from MRI scans of male C57BL/6 mice. All non-brain metabolic regions were removed by loading the scans together with a brain mask file created from the brain template and using the PMOD manual co-registration functions to eliminate all pixel values outside the brain area. After PMOD preprocessing, images were analyzed with MATLAB (version 7.7.0, R2008b, https://www.mathworks.com/) using the SPMMouse package (version 1.0, https://github.com/neurospin/spmmouse) that worked within SPM (version 5.0, Wellcome Department of Imaging Neuroscience, Institute of Neurology, London, UK, https://www.fil.ion.ucl.ac.uk/spm/software/spm5/). All images were registered using the *realign* and *reslice* function twice, first aligning to a template image and then aligning to the mean of all images, and then smoothing was performed. The resulting images were used for further analysis.

### Region-of-interest analysis

It was done using MATLAB (version R2024a, MathWorks, Inc., Natick, MA, USA, https://www.mathworks.com/products/new_products/release2024a.html). We generated templates for all regions-of-interest by taking the MRI templates to which the scans were aligned, and then drawing masks into coronal slices for each region based on the Franklin and Paxinos anatomical atlas^90^. The preprocessed and registered images were loaded into MATLAB and normalized to the mean value for each mouse. Then, masks were applied to each scan to obtain SUVs for each hemisphere of each coronal slice that was drawn. Heatmap images were created for the SUVs for each region in MATLAB using the JET colormap and overlaying onto the corresponding coronal slices downloaded from the Allen Brain Map Reference Atlas^53,54^ for visualization purposes. For each mouse, we subtracted the F1 SUVs from the F3 SUVs of each hemisphere within each slice and represented these values as ΔSUVs.

### Machine learning classifiers

We used the Statistics and Machine Learning Toolbox in MATLAB (version R2024a, MathWorks, Inc., Natick, MA, https://www.mathworks.com/products/statistics.html). As input data, we used the ΔSUVs obtained from the region-of-interest analysis. For each iteration of the loop, one mouse was isolated from the rest of the dataset to be used as test data. The remaining mice were then used as the training and validation data to train the model and optimize the hyperparameters. We used the *fitclinear*, *fitcsvm*, and *fitcensemble* functions to create the model for each loop. We then applied the *predict* function in MATLAB, using the trained model to predict the class of the test data. This entire process was repeated for each iteration of the leave-one-out cross validation loop.

### ORT analysis

Preprocessed and registered images from CON mice from F1 and F3 were used as the two conditions for this approach, which was implemented with a custom script in MATLAB. The Akaike information criterion for each PC (and combination of PCs) was applied, and the lowest score for this value was used for further analysis. The result was bootstrapped 500 times, and a permutation test with 500 iterations was performed. The bootstrapped voxel weights were shown overlaid onto an MRI template to which the PET scans had been aligned. This was displayed using FSL (version 6.0.1, FMRIB Software Library, Analysis Group at the Wellcome Centre for Integrative Neuroimaging, Oxford, UK, https://fsl.fmrib.ox.ac.uk/fsl/docs/#/)^91^. The nodal expression was then evaluated for each mouse, and the difference in expression from F1 to F3 was calculated.

### Statistical analysis

It was carried out using Origin Pro (version 2024, OriginLab, Northampton, MA, USA, https://www.originlab.com/) and Statistics and Machine Learning Toolbox in MATLAB (version R2024a, MathWorks, Inc., Natick, MA, https://www.mathworks.com/products/statistics.html). Nested datasets were analyzed using mixed model ANOVA. Datasets with normal distributions were assessed for statistical significance using Student’s t test, while datasets with non-parametric distributions were tested using Mann-Whitney U test or Kolmogorov-Smirnov test. *P* < 0.05 was used as the cutoff for statistical significance.

## Electronic supplementary material

Below is the link to the electronic supplementary material.

Supplementary Material 1

## Data Availability

The authors declare that all the datasets supporting the findings of this study are available within the manuscript and its supplementary information files. The MATLAB code used to generate SUVs and perform machine learning classification is available at: https://github.com/HuertaLab/20240730_beta_PET (10.5281/zenodo.13136023).
